# Potential role of gut microbiota in prostate cancer: immunity, metabolites, pathways of action?

**DOI:** 10.3389/fonc.2023.1196217

**Published:** 2023-05-17

**Authors:** Cheng Zha, Zheng Peng, Kunyuan Huang, Kaifa Tang, Qiang Wang, Lihua Zhu, Bangwei Che, Wei Li, Shenghan Xu, Tao Huang, Ying Yu, Wenjun Zhang

**Affiliations:** ^1^ Department of Urology, The Affiliated Hospital of Guizhou Medical University, Guiyang, China; ^2^ Department of Urology & Andrology, The First Affiliated Hospital of Guizhou University of Traditional Chinese Medicine, Guiyang, China

**Keywords:** prostate cancer, intestinal microorganisms, the gut microbiome, microbial metabolites, immune, tumor marker

## Abstract

The gut microbiota helps to reveal the relationship between diseases, but the role of gut microbiota in prostate cancer (PCa) is still unclear. Recent studies have found that the composition and abundance of specific gut microbiota are significantly different between PCa and non-PCa, and the gut microbiota may have common and unique characteristics between different diseases. Intestinal microorganisms are affected by various factors and interact with the host in a variety of ways. In the complex interaction model, the regulation of intestinal microbial metabolites and the host immune system is particularly important, and they play a key role in maintaining the ecological balance of intestinal microorganisms and metabolites. However, specific changes in the composition of intestinal microflora may promote intestinal mucosal immune imbalance, leading to the formation of tumors. Therefore, this review analyzes the immune regulation of intestinal flora and the production of metabolites, as well as their effects and mechanisms on tumors, and briefly summarizes that specific intestinal flora can play an indirect role in PCa through their metabolites, genes, immunity, and pharmacology, and directly participate in the occurrence, development, and treatment of tumors through bacterial and toxin translocation. We also discussed markers of high risk PCa for intestinal microbiota screening and the possibility of probiotic ingestion and fecal microbiota transplantation, in order to provide better treatment options for clinic patients. Finally, after summarizing a number of studies, we found that changes in immunity, metabolites.

## 1 Introduction

### 1.1 Current Status of prostate cancer

Prostate cancer (PCa) is the second most common malignant tumor in men and the fifth largest cause of tumor-related death in the world (1). There are about 800,000 new cases of PCa and 300,000 deaths worldwide every year (2). The risk factors for PCa include genetic history, family history, diet, environment, African population, and elderly population (3–5). The incidence of PCa, according to global cancer statistics for 2020, varies by region and ranges from 6.3 to 83.4 per 100,000 people, with the highest rates found in Northern Europe, Western Europe, the Caribbean, Australia/New Zealand, North America, and Southern Africa, and the lowest in Asian nations (6). However, the pathogenesis and development of PCa are still unclear. One of the accepted explanations is the effect of androgen stimulation and the defect of prostate cell apoptosis (7). When PCa progresses to an advanced stage, it is usually treated with surgery or androgen deprivation therapy (ADT), but almost all patients develop resistance to ADT, which inevitably leads to castration-resistant prostate cancer (CRPC) with a worse prognosis (8). ADT is a significant PCa treatment, but there is no proof that it takes other PCa risk factors, like bacterial infection, environmental changes, or inflammatory effects, into account when planning a treatment plan (9). More and more evidence shows that microflora is involved in the microenvironment of tumors and may play a role in tumorigenesis (10). The gut microbiome can influence the growth and progression of PCa through its derived metabolites, suggesting the existence of a gut-prostate axis (11). For example, another source of androgen may be provided by intestinal microflora, which promotes the progress of PCa (12).

With the increasing incidence of PCa, in order to understand the potential contribution of intestinal microbiota and microbial metabolites to the emergence of PCa, as well as novel approaches for the prevention and treatment of PCa based on intestinal microbiota, this review examines studies on PCa and intestinal microbiota.

### 1.2 The gut microbiome and PCa

The human gut is home to trillions of microorganisms that form a complex community of bacteria, protozoa, fungi, and viruses, which together make up the intestinal microbiota (13). 99% of the total microorganisms in the human body are located in the intestinal tract. Intestinal microbiota not only plays an irreplaceable role locally, but also people pay more and more attention to the distal effect (14). According to their level of pathogenicity, the bacteria in the gastrointestinal tract can be categorized into three groups: pathogenic bacteria, possibly harmful bacteria, and helpful bacteria. When helpful bacteria are present, they can prevent the growth of harmful bacteria, promote the creation of vitamins, inhibit the breakdown and fermentation of food components, and stimulate the immune system and food tolerance. The pathogenic bacteria effects are the body’s food intolerance, infection, and inflammation. Potential pathogen species belong to the normal microbial community in the gut, such as Escherichia coli, Enterobacteria, Streptococci, Enterococci, and Bacteroides. When changes occur in the body’s gut that cause these microbes to be present in large numbers, they can cause disease (15). The imbalance of intestinal flora can lead to flora imbalance, and long-term flora imbalance may lead to obesity, type 2 diabetes, insulin resistance, hyperlipidemia, hypertension, metabolic dysfunction, inflammation, and cancer (16).

Intestinal microflora is affected by a variety of external factors, including environmental, dietary, and regional changes, and is involved in all stages of cancer, including initiation, progression, treatment outcomes, and adverse reactions (17–20). The presence of a tumor may lead to an increase in microbial diversity at the tumor site and a decrease in the diversity of gut microbiota (21), and this change may be a precursor to the occurrence and development of cancer, or even directly lead to the occurrence of cancer. Recent years have seen a rapid development of bacterial 16s ribosomal RNA genome sequencing and shotgun sequencing as human microbiome analytical tools. Future options for treating cancer, particularly PCa, are anticipated to include the microbiome because it plays a significant role in immune system control (22, 23). The set of genes of intestinal bacteria is defined as a functional estrogen genome, and its products can metabolize estrogen. The potential effect of individual intestinal microflora on estrogen group is also considered to be a possibility. Since there is an established correlation between estrogen levels and PCa risk, the metabolic effects of intestinal microbiota are associated with PCa risk (24). With the growing understanding of the role of microbes in the process of cancer development, several studies have been conducted to investigate the link between specific gut microbes and PCa. There were significant differences between PCa patients and healthy patients. Compared with healthy patients, the intestinal microbiota of PCa patients showed an increase in α-diversity and structural changes (25–27). Golombos et al. compared rectal swab samples from PCa patients with healthy patients and found a higher relative abundance of *Bacteriodes massiliensis* and a decrease in the number of bacteria that produce folic acid and biotin (26). Liss et al. found that *Bacteroides* and *Streptococcal* were significantly enriched in PCa patients compared with non-PCa patients (25). Matsushita et al. enrolled 152 Japanese men who underwent prostate biopsies and divided them into two groups: the high-risk group and the low-risk group. They found that the relative abundance of *Rikenellaceae*, *Alistipes*, and *Lachnospira* increased significantly in the high-risk group, and all of these bacteria produced short-chain fatty acids (28). Smith included the fecal microflora of 22 patients with overweight breast cancer or PCa (BMI > 25 kg/m2) and 22 controls. It was found that the β-diversity index of PCa was significantly different from that of the control group, and the abundance of *Tissierellaceae*, *Lachnospira*, and *Ruminococcaceae* was higher (29).

The decline in testosterone levels leads to harmful changes in the intestinal microbiota. This ecological imbalance may lead to increased weakness and an increased risk of adverse consequences in patients with PCa. Kure included 24 patients treated with ADT. It was found that the α-diversity and β-diversity of intestinal microbiota and the relative abundance of *Gammaproteobacteria*, *Proteobacteria*, *Pseudomonas*, and *Pseudomonadale* decreased significantly at 24 weeks after ADT (30). Joseph et al. performed 16SrRNA gene analysis on the fecal microflora of 86 PCa patients (56 ADT, 30 underwent prostatectomy) and found that the diversity was significantly decreased in the ADT group. There were significant differences in β diversity among groups. In the ADT group, the relative abundance of *Ruminococcus* and *Bacteroides* was higher, while *Lachnospira* and *Roseburia* decreased. In the ADT group, the ratio of *Firmicutes* to *Bacteroidetes* was also lower (31). Liu et al. analyzed the fecal microflora of 21 patients with hormone-sensitive prostate cancer (HSPC) and CRPC who received ADT by using the sequence of 16S rRNA gene amplifiers. It was found that with the increase in abundance of several bacteria, including *Bacillus* and *Ruminococcus*, the intestinal microflora in CRPC changed significantly (32).

Patients with colon cancer, pancreatic cancer, and melanoma patients can take oral broad-spectrum antibiotics to inhibit the growth and metastasis of their tumors (33). However, oral antibiotic therapy has the opposite effect in PCa, One of the external elements that contribute to ecological issues is antibiotic use, which is thought to be the primary cause of changes in the species of gut microbes that may be temporary or permanent. It has been demonstrated that the risk of numerous malignant tumors, including PCa, is somewhat increased by the use of antibiotics (34). Boursi et al. hypothesized that antibiotics can cause changes in intestinal bacterial diversity and lead to chronic infection. They found in 27212 PCa patients and 105940 volunteers that the use of penicillins, quinolones, sulfonamides, and tetracyclines moderately increased the risk of PCa (35). Tulstrup et al. hypothesized that alterations in intestinal permeability may be connected to antibiotic-induced changes in microbiota. For 10–11 days, they administered amoxicillin, cefotaxime, vancomycin, metronidazole, or water to 60 Wistar rats. It was discovered that the three antibiotics significantly altered bacterial α-diversity, composition, and cecal short-chain fatty acids. The general decrease of intestinal microbial diversity and the increase of *Proteobacteria* relative abundance were observed (36). Matsushita et al. gave PCa mice on a high-fat diet (HFD) an oral antibiotic mixture and found that the number of *Rikenellaceae* and *Clostridiales* increased and the composition of intestinal microflora was significantly different from that of the control group, which inhibited the proliferation of PCa cells and decreased the expression of Igf1 and the level of circulating insulin-like growth factor-1 (IGF1) in the prostate (37).

Diet is closely related to the distribution of intestinal microflora and the construction of intestinal microbial structures. Intestinal microflora also affect the absorption, digestion, and metabolism of food (38, 39). Studies have shown that HFD increases *Anaerobion* and *Bacteroides *(40), transforming a low-fat, plant polysaccharide-rich diet into a high-fat/high-sugar “western” diet, β-diversity changes significantly after a week and changes the representativeness of metabolic pathways in the microbiome (41). The number of *Ochrobactrum* overexpressed in prostatic fluid of PCa patients was also significantly increased in HFD mice (42).In a study of HFD mice, HFD promoted PCa growth through histamine signal transduction in mast cells, and changes in microflora in mouse feces promoted histamine biosynthesis and increased the growth of inflammatory cancer cells (43).

To sum up, compared with healthy patients, there are significant differences in intestinal microflora in patients with PCa, ADT treatment, antibiotic treatment, and HFD. Specific changes in gut microbes may play a role in different types of PCa, which can further explore the role of different intestinal microorganisms in the pathogenesis of PCa, **Table 1** summarizes the studies of specific microbes and PCa risk.

**Table 1:** Specific gut microbes and prostate cancer.

Study	Samples	Findings
David M. Golombos (26)	Fecal samples from 20 patients with benign prostatic hyperplasia and prostate cancer	Bacteroides massiliensis was more abundant in prostate cancer cases
Michael A.Liss (25)	Rectal swab samples from 105 patients undergoing prostate biopsy	Bacteroides and Streptococcus species were significantly enriched in prostate patients
KS Smith (29)	Fecal samples from 22 overweight breast or prostate cancer patients compared with 22 controls	Tissierellaceae, Lachnospiraceae and Ruminococcaceae were significantly increased
Akimasa Kure (30)	Fecal samples from 24 prostate cancer patients treated with ADT	The relative abundance of Pseudomonadales, Pseudomonas, Proteobacteria, and Gammaproteobacteria has decreased in the aftermath of the ADT.
Joseph K. M. Li (31)	Fecal samples from 86 PCa patients (56 receiving ADT and 30 undergoing prostatectomy	The relative abundance of Ruminococcus and Bacteroides was higher in the ADT group, while Lachnospira and Roseburia were reduced
Yufei Liu (32)	Fecal microbiota in 21 HSPC and CRPC patients receiving ADT	The abundance of Bacillus and Ruminococcus increased
Monica Vera-Lise Tulstrup (36)	Fecal samples were obtained from 60 Wistar rats	The relative abundance of Proteobacteria increased after antibiotic exposure
Makoto Matsushita (37)	Fecal samples from Prostate-specific Pten knockout mice subjected to HFD and orally fed an antibiotic mixture	Increased numbers of Rikenellaceae and Clostridiales were observed
Weibo Zhong (168)	Fecal samples from C57BL/6J male mice	The number of Proteobacteria increased significantly after antibiotic exposure

## 2 Pathways of action of the gut microbiota on PCa

Manzoor et al. divided the relationship between microbiota and cancer into three levels: primary (interaction in the tumor proximal microenvironment), secondary (interaction between tumor and microbial community with general compartment tissue or organ system), and tertiary (interaction between tumor and remote microbial community). The main interaction is defined as the direct relationship between the tumor and the microflora in the local tumor microenvironment (44). Intestinal microbiota are not only directly related to local intestinal diseases such as inflammatory bowel disease and colorectal cancer (CRC) (45, 46), but also closely related to the pathogenesis of systemic diseases such as liver or nervous system diseases, which indicates the existence of an intestinal-liver axis and an intestinal-brain axis (47, 48). However, PCa does not have direct contact with the intestinal tract, and the role of intestinal microflora in it is still being explored. The possible factors of the intestinal microbiome in promoting the occurrence and development of PCa are related to the risk factors (obesity and inflammation) affected by the fecal microbiome and may directly cause distant effects in organs such as the prostate (25), Carcinogenesis can also be indirectly induced by metabolites of intestinal microorganisms (such as fatty acids and polyamines) in distal organs (49).

### 2.1 Indirect pathway of action

#### 2.1.1 Metabolites

##### 2.1.1.1 Short chain fatty acids/Growth factor-1

Short chain fatty acids (SCFA) are the main metabolites of the intestinal microbiota, including propionate, acetate, and butyrate, which play an important role in physiology (50). Intestinal microflora produces large amounts of SCFA from indigestible and fermentable carbohydrates, including dietary fiber (51). The change in intestinal structure changes the level of SCFA, which affects cell adhesion, cytokine production, chemotaxis, immune cell recruitment, and apoptosis (52).Intestinal microorganisms can regulate PCa through SCFA. It was found that *Alistipes* and *Lachnospira* producing SCFA were significantly increased in the intestinal microflora of patients with high-grade PCa, suggesting that SCFAs may be the promoter of PCa (28). Supplementation of SCFA increases systemic and local insulin-like IGF1 in the host prostate. IGF1 promotes tumor growth by activating local prostate MAPK and PI3K signal transduction, revealing the possible existence of the intestinal microbiome-IGF1- prostate axis (37). Antibiotics can reduce the SCFA in the feces of mice, and mice fed with a HFD can restore the level of serum IGF-1 and cancel the inhibition on the growth of PCa (53). IGF1 can also directly promote the proliferation of PCa cell lines DU145 and 22RV1 *in vitro (* 37),The incidence of PCa and the risk of PCa-related mortality are also significantly increased in acromegaly patients with systemic high growth hormone and IGF-1 levels, indicating that IGF-1 plays a positive role in the development and progression of PCa (54).

##### 2.1.1.2 Testosterone

Many disorders affecting male patients, including metabolic syndrome, PCa, and delayed hypogonadism syndrome, are heavily influenced by testosterone (55, 56). Intestinal microflora also plays a role in testosterone production. Makoto Matsushita et al. took rectal swab samples from 54 Japanese men with negative prostate biopsies and sequenced the 16S rRNA gene to analyze the intestinal microflora. It was discovered that the quantity of *Firmicutes* positively linked with the level of serum testosterone, and that the quantity of *Firmicutes* had no bearing on host variables (age, body mass index, triglycerides, and total cholesterol) (57).Another study found that there was a significant correlation between the number of *Acinetobacter*,*Ruminococcus*,*Dorea*, and *Megamonas* and serum testosterone levels in men, with high levels of estradiol having more *Bacteroides* and fewer *Firmicutes* than women with low levels of estradiol (58). Compared with HSPC patients, the number of *Ruminococcus* in the intestinal microflora of CRPC patients increased. In patients with CRPC, *Ruminococcus* is associated with a poor prognosis. *Ruminococcus* can convert pregnenolone and hydroxypregnenolone into downstream metabolites of this pathway, including dehydroepiandrosterone and testosterone (59). *Ruminococcus* can also promote the progression of PCa by up-regulating the expression of LPCAT1 and DNA repair proteins (60). *Clostridium scindens ATCC 35704*, *Clonorchis sinensis*, and *Propionibacterium lymphoid* are located in the intestinal microbiota. Their *DesAB* genes encode steroid 17,20-lyase, which can convert cortisol into C19 androgen by splitting side chains, which can be further metabolized by host tissue and the microbiome to form effective 11-oxygen-androgen, while *DesAB*-expressing microorganisms may be neglected sources of androgen *in vivo* and may lead to various disease states, such as PCa (12).

##### 2.1.1.3 Estrogen

Estrogen may be involved in the occurrence and development of PCa (61), so intestinal microbes that cause high levels of estrogen in the body may make men vulnerable to PCa. Normal prostate stem cells express estrogen receptor-α (ER- α), estrogen receptor-β (ER- β) and G protein-coupled receptors. The activation of ER- α is considered to be the cause of PCa. ER- α participates in the transformation of epithelial cells into mesenchymal cells and is closely related to the occurrence and development of tumors. In the mouse model, the bone shape of osteoblasts is inhibited by the ER- α gene knockout model and the ER- α antagonist (62). Plottel and Blaser assume that the collection of genes from intestinal bacteria is a functional estrogenic genome whose products can metabolize estrogens. In particular, the intestinal microbiota contains β-glucuronidase and β-glucuronide. When the intestinal microbiota is maladjusted, it can promote the uncoupling and recycling of estrogen by secreting these two enzymes and bind to the estrogen receptor for the development of PCa. The activity of these two enzymes can help reduce the risk of PCa (24). By activating polycyclic aromatic hydrocarbons (PAHs), estrogen produces carcinogenic metabolites such as diol epoxides and free radical cations, which react with DNA to promote carcinogenesis. The functional estrogen genome of Plottel promotes this estrogenic mechanism to increase the rate of carcinogenesis (63). Therefore, we should pay attention to the increase of serum estrogen in patients with intestinal microbiota disorders and formulate a new treatment strategy.

##### 2.1.1.4 Folic acid

The intestinal microflora is considered one of the sources of folic acid. They cannot be synthesized by the human body and must be absorbed through the intestines from diet or intestinal flora. Bifidobacterium and Lactobacillus, which produce folic acid, may be the supplementary endogenous sources of folic acid and increase the level of folic acid in the human body (64). Folic acid is closely related to PCa, and the expression of the folate receptor is increased in PCa (65). Folic acid plays a key role in the synthesis of nucleotides needed for tumor cell proliferation and DNA methylation. The change in prostate methylation pattern is an important event in PCa (66). Folic acid is a transmembrane carboxypeptidase with hydrolase activity that can decompose prostate specific membrane antigen, which is overexpressed in almost all PCa, and the tissue level of this protein is positively correlated with a higher grade, a higher Gleason score, and disease recurrence (67, 68). Unmetabolized folic acid is associated with reduced cytotoxicity of natural killer cells, which may protect the clearance of malignant cells (65). When Liss et al. compared PCa patients with healthy people, they found that the number of bacteria related to carbohydrate production increased in prostate patients, while the number of bacteria related to folate, biotin, and riboflavin decreased (25). In a controlled randomized trial of 643 men, Figueiredo et al. found that the estimated probability of being diagnosed with PCa within 10 years was 9.7% in those with high folic acid levels and 3.3% in the placebo group. Men who randomly took folic acid supplements were 2.6 times more likely to be diagnosed with PCa than those in the placebo group (69).

##### 2.1.1.5 Phenylacetylglutamine

Phenylacetylglutamine (PAGln) is a phenylalanine metabolite produced by the metabolism of intestinal microflora, which activates α-adrenergic and β-adrenergic receptors through adrenergic receptors (70). Reichard included baseline serum samples from 173 patients with lethal PCa and 519 patients with non-fatal PCa into a case-control design and found that adrenergic compounds produced by metabolism mediated by intestinal microflora were associated with an increased risk of fatal PCa, while β-adrenergic blockade may be another target for reduced risk of PCa (71).

These studies have shown that specific intestinal flora plays a crucial role in the progression of PCa indirectly through its metabolites (short-chain fatty acids, testosterone, estrogen, folic acid, and PAGln), and the metabolites of intestinal flora indirectly affect the occurrence and development of PCa. As for how to play a role in the prevention and treatment of PCa by influencing the production of metabolites, its carcinogenic mechanism and action pathway are worthy of further exploration.

#### 2.1.2 Immunization

The host’s immune system can affect the microbial ecosystem and have an impact on the fecal metabolic content, even if the external environment has a significant role in developing the gut microbiome (72). The following are the effects of intestinal microbiota on tumor immune response: activation of regulatory T cell proliferation and differentiation; induction of IgA expression; and the influence of antimicrobial peptide expression, microbial metabolism, systemic inflammation regulation, and bacterial translocation (73). The intestinal mucosal immune system is made up of gut epithelial lymphocytes, lamina propria lymphocytes, collecting lymph nodes, and other components. The bulk of intestinal epithelial lymphocytes are CD3+ T cells, while B cells and NK cells are in the minority. T and B cells make up the vast majority of lymphocytes in the lamina propria. and regulatory T cells are characterized by the expression of CD4, CD25, and Foxp3. Additionally, the generation of the anti-inflammatory cytokines transforming growth factor-β (TGF-β) and IL-10, which reduce intestinal inflammation (74). There are four components to the defensive mechanism between the intestinal immune system and the mucosa: 1) The microbial barrier is the first element; the microbiome is found in the mucous layer’s upper layer. These symbiotic bacteria have the ability to inhibit the colonization of pathogens, create metabolites or immune signaling-regulating components, and support immunological homeostasis (75–77); 2) The mucus-based chemical firewall that covers the gut epithelium is the second firewall. Through the production of mucus by goblet cells in the epithelium, the release of antimicrobial peptides by the epithelium, and the production of mucosal IgA by dendritic cells (DCs) in the gut, mucus regulates contact between symbiotic bacteria and epithelial cells and protects the epithelium from symbiosis (76); 3) The single-celled epithelial cell layer, which serves as a physical barrier, makes up the third element. In addition to helping with food absorption, the intestinal epithelium serves as a physical barrier against disease invasion and the movement of symbiotic microbes outside the gut (78); 4)The final component is the immune barrier, which contains specialized immune cells (macrophages, DCs, and lymphocytes). Macrophages and DCs are DCs distributed in the lamina propria and mesenteric lymph nodes. Using the intestinal lumen as a source, DCs are efficient antigen-presenting cells that deliver microbial antigens to T lymphocytes in mucosal tissue. Leads to T cell subset development and activation (Th1, Th2, Th17, or Treg), which, along with macrophages, serves as a “bridge” between innate and acquired immune responses (79, 80).The intestinal mucosal immune system not only produces immune tolerance to symbiotic bacteria and food antigens but also produces a strong immune response to pathogenic bacteria and maintains a balanced response to infection (36). By controlling innate and adaptive immune responses, intestinal flora can keep the gut environment balanced. However, specific alterations in the intestinal microflora’s makeup may stimulate the mucosal immune system, cause chronic inflammation and mucosal damage, and encourage an intestinal mucosal immunological imbalance, which in turn promotes the growth of tumors.

##### 2.1.2.1 Innate immunity

The cells of the intestinal flora that regulate innate immunity include monocyte/macrophage, DC, granulocytes, NK cells, and NKT cells. Pathogen-associated molecular patterns(PAMPs) is a conservative structure of pathogens, such as fat polysaccharide, peptidoglycan, lipoprotein, nucleic acid, and so on, with corresponding PAMPs pattern recognition receptors (PRRs), such as toll-like receptors (TLRs) and NoD-like receptors (NLRs), identify conserved structures of microorganisms to obtain innate immune cells and epithelial cells (81), and turn on particular signalling pathways that encourage inflammation, tumour growth, or combat cell death. The stimulation of NF-B signalling and the development of bacterial communities are both crucially dependent on NOD2. Mutations that cause NOD2 malfunction may result in an alteration of intestinal flora and a higher risk of CRC. And NoD1-mediated recognition of the MesoDAP peptidoglycan component in Gram-negative bacteria in the gut microflora has been shown to initiate neutrophils to kill bacterial pathogens (82). The control of intestinal homeostasis is greatly influenced by intestinal TLRs. Pro-inflammatory cytokines, including interleukin and tumour necrosis factor α, are produced when TLRs are activated. Through several growth factor receptor ligands (amphiregulin and hepatocyte growth factor), TLRs may potentially encourage the proliferation of cancer cells to have both local and distant impacts (83). In addition, the intestinal microbiome appears to act on intestinal DC directly or indirectly through epithelial cells, affecting the phenotype and activity of DC (34,102). This stable DC phenotype promotes non-inflammatory responses such as FoxP3+Treg induction and IgA secretion, allowing the host and intestinal mucosal microorganisms to maintain homeostasis (84, 85). IL-10 produced by enteric macrophages inhibits intestinal inflammation by maintaining FOXP3 expression in Treg cells and inhibits IL-12 and tumor necrosis factor-production in enteric myeloid cells by activating the transcription factor STAT3. Thus, mice with IL-10 deficiency and specific STAT3 mutations in myeloid cells (LysM-cre;Stat3^flox/flox^ mice) spontaneously produce intestinal inflammation (86). In prostatitis, it is characterized by the infiltration of macrophages, neutrophils, and lymphocytes, which release reactive oxygen species, reactive nitrogen, and proinflammatory cytokines, leading to DNA damage, cell damage, and cell death. Persistent chronic inflammation leads to proliferative inflammatory atrophy. Prostatic intraepithelial neoplasia and PCa are the ultimate results of chronic inflammation (87).

##### 2.1.2.2 Adaptive immunity

Both innate and adaptive immune responses are influenced by the symbiotic flora of the immune system. For example, mice without bacteria have an undeveloped adaptive immune system (88). Differentiation of Peyer’s patches(PPS), CD4+ T cells in the lamina propria, IgA-producing B cells, and intestinal epithelial lymphocytes have been associated with intestinal adaptive immunity induced by the intestinal microbiome. It is necessary to maintain the stability of the intestinal environment, the integrity of the intestinal barrier, and immune tolerance to symbiotic bacteria (89). Certain metabolites are essential for controlling the activity of adaptive immune cells, particularly CD4+ T and B lymphocytes. Additionally, in the gut and other distant organs, microbial imbalance makes the host more vulnerable to a variety of immunological, inflammatory, and allergy illnesses (90, 91). For instance, the microbiota promotes the development of cancer, which is directly influenced by gut Th1 and Th17 cells. Th1/Th17 balance has been shown to be associated with prognosis in patients with CRC, and one study found that Th17 cells produced a higher proportion of Th1 and Th17 cells as well as the cytokines IL-17A, IL-22, and IL-23A when fed stool from patients with CRC in regular mice (92, 93). In PCa tissues, the abundance of microbiome is highly correlated with the expression of Treg. These cells inhibit the activation and proliferation of effector T cells and ultimately inhibit the host immune response (94). Inflammation and carcinogenesis may trigger barrier failure, but barrier failure also promotes inflammation and cancer, creating a feedback loop (83), in which the intestinal barrier’s breakdown results in bacterial translocation and the emergence of a systemic inflammatory response (95). Several bacteria induce immunity during tumor development. Cytotoxic immune cells (cytotoxic T lymphocytes) are required for *Bifidobacterium*, *Bacteroides thetaiotaomicron*, and *Bacteroides fragilis* to enhance antitumor cytotoxic T cell immunity. *Fusobacterium nucleatum* promotes tumor growth by inhibiting T cell activity, which is associated with survival and antitumor therapeutic efficacy (96). Terrisse recently conducted a prospective study that included a mouse model of PCa and fecal and blood samples from 65 patients with HSPC and CRPC. It was found that ADT increased thymocyte count and output in normal mouse models. The response of PCa and ADT implantation in mice with T lymphocyte loss or thymus loss was lower than that in normal mice. Oral antibiotics destroyed the diversity of intestinal flora and decreased the efficacy of ADT. PCa also reduced the relative abundance of *Akkermansia muciniphila* (*A.muciniphipla*) in the intestine, which could be reversed by ADT. In addition, compared with the HSPC control group, the intestinal microflora of CRPC patients had a significant correlation with the abundance of thymic transitional cells, indicating a functional relationship between the intestinal ecosystem and the thymus (97).

##### 2.1.2.3 Regulation of the immune response

In specific cases, cytokines regulate cell growth differentiation and effects by binding to corresponding receptors, regulate immune responses, and play a role in the development of many diseases, including inflammation. For example, TGF can be used by *Staphylococcus aureus* and *Streptococcus Group A* to promote the growth and spread of tumour cells (98–100). Intestinal microflora can establish a pro-inflammatory or anti-tumor environment by regulating the function of host physiological and immune cells (101), and its changes can affect the inflammation of distal organs (102, 103). Interleukin B, TGF-β, vascular endothelial growth factor, tumor necrosis factor, etc. are released in response to intestinal dysregulation. Both growth factors and cytokines cause inflammation, which impairs differentiation by preserving the presence and expansion of undesirable cells (104). If inflammation continues, cancer will develop as a result of the ongoing spread of inflammatory signals, inhibited apoptosis, and elevated amounts of growth factors (105). *Verrucommicrobi*, to which *A.muciniphipla* belongs, promoted the inflammation of intestinal microflora in mice, and its abundance increased with the increase of chemokines and cytokines in mucositis induced by 5-fluorouracil. Extracellular vesicles from *A.muciniphipla* can increase the number of M1-like macrophages and secrete more inflammatory cytokines (10). Poutahidis et al. found that *Helicobacter hepaticus* was found in the intestinal flora of Apc^Min/+^ mice, which significantly increased the incidence of prostatic intraepithelial neoplasia and PCa (106). *Helicobacter hepaticus* infection can cause systemic elevation of pro-inflammatory cytokines (eosinophil chemokine, IL-3, tumor necrosis factor-α and IL-1α) and enhance prostatic intraepithelial neoplasia and microinvasive carcinoma (10).

Gut bacteria have a direct influence on immune response, and changes in the composition and number of microbiota may influence local immune response. We now know that changes in the types of bacteria in the gut can also lead to immune changes in distant organs that can cause cancer.

#### 2.1.3 Genetic phenotype

In the past, the interaction between intestinal flora and host function was not recognized. The number of microbial cells carried by the human body is 10 times that of the total number of cells in the human body, and the genetic information is 150 times that of the human genome. Therefore, there are many complex interactions between microbes and their hosts (107). The metabolic activity of intestinal microbiota is very important to maintain the homogeneity and health of the host. Although the existence of microbiota is very important, changes in its composition lead to metabolic changes, which may lead to changes in the host phenotype. Lynch et al. proposed the “common ground” hypothesis: genetically susceptible hosts disrupt intestinal microbiota due to environmental factors such as diet or chronic infection, resulting in polygenetic changes that result in host diseases such as cancer (108). It has been found that the gene toxin Colibactin can be secreted by Escherichia coli, which induces the appearance of senescent cells and promotes tumor growth by secreting growth factors (109). In addition, when FMT containing CRPC feces was injected into mice, it was found that the expression of DNA-PKcs, RAD51, and LPCAT1 in the prostate tissue of mice was positively correlated with the malignant degree of PCa (60). *Sphingosine 1-phosphate receptor 2* (*S1PR2*) is a gene mainly regulated by a HFD, which is associated with abundantbody mass index, Lactobacillus, a low BMI and aggressive characteristics in patients with PCa (110). Additionally, *S1PR2* expression is seen in host endothelial cells and tumor-infiltrating bone marrow cells, where it suppresses the expression of vascular endothelial growth factor and matrix metalloproteinase-9 activity to prevent tumor angiogenesis and tumor growth (111). HDF can promote the growth of Lactobacillus, reduce the number of Bacteroides, and change the composition of intestinal microorganisms, which is closely related to the progression of obesity (112). Obesity caused by HFD can lead to chronic systemic inflammation, activate signal pathways, and promote the progression of PCa through immune system-related mechanisms, including activating a series of chemical signaling pathways such as IL6/pSTAT3 or MCP-1/CCR2, inhibiting the tumor suppressor gene PTEN to induce the growth of PCa (113), and inducing local IL-6 upregulation in immune cells and MDSC, enhanced MYC transcriptional programming through metabolic changes increases histone hypomethylation in the promoter region of the MYC regulatory gene (114). HFD can also increase the number of Lgr5+ intestinal stem cells and promote tumor formation by activating and enhancing the peroxisome proliferator (PPAR-d) signal, making intestinal stem cells lose the tumor suppressor APC (115). Inflammation is a risk factor for tumorigenesis and progression. Inflammation may trigger cell repair, angiogenesis, and tissue repair cascades to a greater extent by affecting cell and genome damage, thus promoting tumor occurrence and progression (116). In addition, inflammation may promote immune cells to release reactive oxygen species and reactive nitrogen, which directly damage cells and DNA, resulting in proliferative inflammatory atrophy, which is considered to be the pathogenic factor of PCa and other cancers (63). Inflammation and HDF are closely related to the change of gene phenotype. The interaction between them and intestinal flora changes the composition of intestinal flora and thus changes the metabolic pathway and absorption, which may result in different responses to PCa.

### 2.2 Direct pathway of action

#### 2.2.1 Bacterial translocation

The process of intestinal microorganisms and their metabolites transferring to mesenteric lymph nodes or portal veins through the intestinal mucosal barrier and entering other organs is called “bacterial translocation” (117). The risk factors include the disorder of intestinal flora (118), the increase in intestinal permeability (119, 120), and the deficiency of immune function (121, 122). Intestinal microbial translocation has been proven to play an important role in the pathogenesis of many diseases, such as pancreatitis (123), and liver cirrhosis (124).

The decrease in the diversity of intestinal microflora, the disorder of flora structure, and the quantitative changes in intestinal metabolites during PCa will eventually lead to intestinal microbial imbalance. Intestinal microbial disorders caused by various factors will damage the integrity of the intestinal wall, and increasing intestinal permeability will lead to intestinal metabolites or bacterial components that can cause disease in the systemic circulation (125, 126). We call this process “bacterial passive translocation.” A frequent indicator of intestinal health is intestinal permeability, bacteria may breach the epithelial barrier as a result of increased intestinal permeability, causing inflammation. Studies have shown that biological involvement in prostate cancer is complex, but it has been determined that epithelial barrier breakage is related to inflammatory changes in the prostate microenvironment and prostate infection, which promotes the development of prostate cancer (127). There is growing evidence that intestinal microflora may play a role in the progression of PCa by affecting intestinal permeability (128, 129). Existing studies have found that when intestinal permeability increases, the passive translocation of bacteria may occur in two main pathways: the paracellular pathway controlled by tight junctions or the specific intestinal epithelial cell pathway and the transcellular pathway controlled by the membrane pump (130). There are a variety of microorganisms that can produce cytotoxins that change the structure of intestinal epithelial cells and lead to passive translocation. *Enterohemorrhagic E. coli*, *Salmonella typhimurium*, *Clostridium perfringens*, *Bacteroides fragilis*, *Vibrio cholerae*, and *rotaviruses* can release virulence to attack intestinal epithelial cells, causing tight junction proteins to destroy and cross the membrane (131, 132). *Enteropathogenic E. coli*, *Helicobacter pylori*, *Clostridium difficile*, and *Pseudomonas aeruginosa* release toxins to increase the permeability of intestinal epithelial cells without changing the structure of tight junctions (133–136). *Shigella flexneri*, *Listeria monocytogenes*, and *Clostridium botulinum* can break the epithelial cell barrier by modifying the actin cytoskeleton (137, 138).

Studies have found that HFD leads to an increase in intestinal permeability, but subsequent antibiotic treatment will further reduce intestinal permeability (139). The destruction of intestinal microflora by metronidazole and other antibiotics led to an increase in intestinal inflammatory tension, an increase in bacterial stimulation of epithelial cells, a change in goblet cell function, and the thinning of the internal mucus layer, indicating the weakening of mucosal barrier function and the increase in intestinal permeability (140). In one mouse model, treatment with penicillin or metronidazole increased the number of *Enterobacter* in the cecum by an average of 1000 times, while treatment with clindamycin increased the number of *Enterobacter* by 100000 times. The average incidence of translocation to mesenteric lymph nodes was 100% after penicillin treatment, 97% after clindamycin treatment, and 62% after nail-file treatment (118). Steffen et al. found that the level of a specific cecal population may be the main factor promoting the translocation of bacteria from the intestine. In the mouse model, the number of *E. coli, P. mirabilis*, and *Klebsiella pneumoniae* in the cecum was significantly correlated with the number of these bacteria transferred to the mesenteric lymph node complex (141). In diseases such as trauma, shock, and heat injury, anoxic injury and ischemia or reperfusion of intestinal epithelial cells greatly disrupt the cytoskeleton, resulting in increased permeability of intestinal epithelial cells and bacterial translocation (142, 143). Immune deficiency is an important factor leading to intestinal microbial translocation. The incidence of intestinal bacterial translocation is 50% in non-thymic mice and only 7.8% in transplanted thymic mice (121). In a normal intestinal environment, intestinal mucus secretes immunoglobulin A (sIgA) to prevent bacteria from adhering to the surface of intestinal epithelial cells, cooperate with complement and lysozyme, and encapsulate invasive viruses, which is an important line of defense to maintain the intestinal immune barrier (144). When immune function deficiency occurs, it leads to a decrease in intestinal mucus sIgA, which greatly promotes the passive translocation of bacteria (145).

When bacteria invade extra-intestinal organs through the intestinal barrier without evidence of a pathological environment or trauma, “active bacterial translocation” occurs. The three basic steps of bacterial active translocation are adhesion, invasion, and actual movement through the intestinal barrier (146). When intestinal epithelial cells are intact, Cossart et al.’s mechanisms for internalizing bacteria into non-phagocytic cells such as intestinal epithelial cells are divided into three categories: 1) Some microbial pathogens usually express a surface protein that binds to eukaryotic receptors and participates in cell matrix or cell adhesion and expression, leading to vacuole formation, actin cytoskeleton rearrangement, and membrane extension receptor binding. Trigger the “zipper mechanism” to phagocytize the pathogen. 2) Other microbial pathogens can express a binding protein that acts as a bridge between bacteria and receptors, and thus transmembrane receptors mediate entry into the cell; 3) Other microbial pathogens do not play a role through the process of adhesion; their secretory systems inject special effectors into cells, directly regulate actin cytoskeleton rearrangement, and eventually form a large phagocytic bag inside the cell (147). In cells, microbial pathogens polymerize actin to provide energy, which makes bacteria move to the plasma membrane and form protuberances and finally form vacuoles through phagocytosis of adjacent cells (147).

After passing through the intestinal barrier, microbial pathogens may enter the systemic circulation mainly through two pathways: through the intestinal venous system into the portal vein or through intestinal lymphoid drainage (130). In pancreatitis, the pathway of bacterial translocation may be hematogenous spread, directly transferred to the peritoneal cavity or retroperitoneal and then to the pancreas, or secondary to the lymph near the pancreas (148, 149). A retrospective study found that bacterial translocation was involved in the biochemical recurrence of PCa. They found that plasma bacterial 16S rDNA levels increased in patients with PCa biochemical recurrence, and the grade of PCA patients was positively correlated with plasma 16S rDNA levels (150). Whether there is a bacterial translocation similar to the pancreatitis pathway in the prostate may be a focus of microbial research in the future.

#### 2.2.2 Toxin translocation

Bacteria themselves do not need to cross the intestinal epithelial barrier to cause disease, but the infiltration and translocation of inflammatory or toxic substances produced by intestinal microorganisms may also lead to body damage, called “toxin translocation” (151). Outer membrane vesicles are regarded as “remote weapons” of bacteria, which can promote the interaction between bacteria and bacteria, bacteria and hosts, and transport bacterial toxins or other virulence factors to host cells to cause disease, making a great contribution to toxin translocation (152). Some studies have proposed the intestinal lymphoid hypothesis, which states that toxic mediators are released from the intestines after intestinal injury and are transported through mesenteric lymph nodes to cause disease, while another study supports this speculation. After blocking mesenteric lymph nodes, the distal organs of mice can be prevented from being damaged by toxin translocation (153, 154).

Toxin translocation most often leads to sepsis and multiple organ dysfunction syndrome (155, 156), but its role in tumors is still being explored. The intestinal tract is the largest pool of endotoxin in the human body. The level of endotoxin in the blood can reflect the damage to the intestinal mucosal barrier and the level of bacterial translocation. The intestine usually absorbs a small amount of endotoxin, but when endotoxin enters the liver through the hepatic portal vein, the liver produces Kupffer cells to clear the endotoxin. When the intestinal microflora is in disorder, it may induce the immune and inflammatory reactions of the intestinal mucosa and eventually lead to an increase in intestinal mucosal permeability and endotoxin translocation. With the progress of the disease, when the level of portal vein endotoxin is too high, it can stimulate liver Kupffer cells to release a series of cytokines, such as tumor necrosis factor, interleukin-1, interleukin-6, free radicals, and so on (157). Dietary factors such as fat, fructose, and alcohol can change intestinal flora and intestinal permeability and make enterogenic toxins pass through the intestinal barrier to activate hepatocytes and overproduce inflammatory cytokines. The pathogenesis is that NF-κB activates B cells, which leads to systemic inflammation and body injury (158, 159). When the intestinal microflora is destroyed or out of balance, the intestinal barrier function may be impaired, thus increasing permeability and thus increasing the chances of leakage of intestinal fluid, macromolecules, white blood cells, toxins, and compounds into the circulation, which may also lead to inflammation (160). The decrease in the diversity of intestinal microorganisms leads to the overgrowth of bacteria and leads to mild systemic inflammation, called endotoxemia, which leads to an overall inflammatory state in many organs and promotes tumor formation (25).

### 2.3 Pharmacological effects

#### 2.3.1 Chemotherapy and immunotherapy

Through a number of processes, such as immunological interaction, diverse metabolism, and altered community structure, intestinal microflora can control the host’s reaction to chemotherapy. The pharmacological effects of some chemotherapeutic drugs and immunotherapy are closely related to intestinal bacteria, such as 5-fluorouracil, cyclophosphamide, irinotecan, oxaliplatin, gemcitabine, methotrexate, anti-PD-L1 therapy, and anti-CLTA-4 therapy (161). James Alexander et al. proposed a framework for how intestinal microflora affect the pharmacological effects of these drugs through important mechanisms: “TIMER,” which represents translocation, immunomodulation, metabolism, enzyme degradation, reduction of diversity, and ecological variation (162).

Immunotherapy is an essential part of the treatment of PCa. Emerging evidence suggests that the gut microbiome influences the response to anticancer treatments by modulating the host immune system. Martina Di Modica et al. studied the role of intestinal flora in the anti-tumor efficacy of immune-mediated trastuzumab and found that intestinal microflora was directly involved in the efficacy of trastuzumab. They concluded that controlling intestinal microflora was the best strategy to improve the efficacy of tumors in the future (163). Ayelet Sivan et al. believed that manipulating the microbiome might regulate cancer immunotherapy. They found that oral *bifidobacterium* controlled tumors better, and the PD-L1 blocking effect played a better role in mice with a higher abundance of *bifidobacterium* (164). Other studies have shown that Mycoplasma hyorhinis can metabolize gemcitabine into inactive metabolites, reducing its anti- PCa efficacy (165). Drug resistance is closely related to abnormal intestinal microflora and antibiotic therapy in patients with advanced PCa during immunotherapy (166). Liu et al. found that Docetaxel (DTX) reduced the diversity of the intestinal microbiota. In addition, intestinal diversity decreased and tumor growth accelerated in mice pretreated with the antibiotic mixture (167). Zhong et al. established a mouse model of intestinal flora imbalance and collected fecal samples from mice for 16S rRNA sequencing. It was found that intestinal microflora disorder after antibiotic exposure led to a significant increase in the number of *Proteobacteria*, intestinal permeability, and lipopolysaccharide in tumors, which promoted the progression of PCa and docetaxel resistance through NF- κ B-IL6-STAT3 axis (168). Marie Vétizou et al. revealed that intestinal flora played a key role in CTLA-4-blocked immunostimulation. They built aseptic mice and mice pretreated with antibiotics and found that they were less effective in anti-CTLA-4 therapy, after the fecal microorganism transplantation of patients containing *Bacillus fragilis*, the effect of anti-CTLA-4 treatment in mice was significantly improved (169).

The diversity and structure of the intestinal microbiota are affected by chemotherapeutic drugs, which in turn affect the efficacy and gastrointestinal toxicity of chemotherapy. Cyclophosphamide is an important anti- PCa drug, and recent studies have shown that its therapeutic efficacy also depends on intestinal microflora. Xu et al. found that cyclophosphamide decreased the proportion of *Bacteroidetes* and increased the proportion of *Firmictutes* in mice (170). Viaud et al. hypothesized that intestinal microflora played a part in shaping the anti-cancer immune response and found that part of the efficacy of cyclophosphamide was due to their ability to change the composition of intestinal microflora and then induce bacterial translocation to stimulate the anti-tumor immune response caused by lymphatic organ infiltration (171). By sequencing the 16S rRNA gene of 28 male stool samples, Montassier et al. found that, compared with the samples collected before chemotherapy, the abundance of fecal samples collected after chemotherapy decreased significantly, containing a small amount of *Firmicutes* and *Actinobacteria*, while the number of *Proteobacteria* increased significantly (172). By establishing a mouse model, Lida et al. found that intestinal microflora regulates the function of myelogenous cells and immune cell response to promote the efficacy of chemotherapeutic drugs, while the disorder of intestinal microflora caused by the introduction of antibiotics reduces the effect of chemotherapeutic drugs, indicating that complete intestinal microflora is needed to regulate immune cell response in the tumor microenvironment (161). Romain et al. established an aseptic cancer mouse model that showed reduced Th17 response and resistance to cyclophosphamide, while the anticancer effect of cyclophosphamide could be restored by oral administration of *Gram-positive Enterobacter (* 173).

#### 2.3.2 Anti androgenic therapy

Androgen deprivation therapy (ADT) is the basis for the treatment of advanced PCa. Intestinal microbes also affect the efficacy of ADT. A recent study found that symbiotic intestinal microflora play a role in endocrine resistance to CRPC by providing another source of androgen, Pernigoni et al. found that *Rumencocci* played a key role in promoting the progression of CRPC. They constructed a PCa model mouse that received castration treatment, and the abundance of *Rumencocci* was significantly increased. After the intestinal flora of mice was removed with antibiotics, the efficacy of ADT was increased, and the progress of CRPC in ovariectomized mice was inhibited. On the other hand, they transplanted intestinal microbes from CRPC patients into PCa mice, which also promoted their PCa progress (59). Liu et al. confirmed that, compared with the intestinal microflora of HSPC patients, the number of rumen cocci in CRPC patients increased significantly (32). Sfanos et al. hypothesized that oral androgen receptor axis-targeted therapy (ATT) may be related to differences in the composition of gastrointestinal microflora. They conducted a cross-sectional study of 30 healthy patients and PCa patients. It was found that the α-diversity of gastrointestinal microbiota was greater in healthy men, and there was a measurable difference in the bacterial composition of the gastrointestinal microbiota in men treated with ATT. The results showed that the abundance of *A.muciniphipla* and *Ruminococcaceae* spp. was higher, which may mean that the changes in gastrointestinal microbiota caused by ATT represent the mechanism of a potential alternative pathway of steroid metabolite production, which in turn affects the treatment and prognosis of tumors (27). Cimadamore et al. showed that both *ruminococcus* and *A.muciniphipla* are involved in steroid biosynthesis, and the relative abundance of *A.muciniphipla* and *ruminococcus* is higher in PCa patients taking ATT, which they believe is more beneficial to the efficacy of anti-programmed death-1 (PD-1) immunotherapy, while the use of antibiotics in patients with *ruminococcus* will increase the risk of progressive PCa (174). Androgen inhibitors are generally used in the treatment of advanced PCa; androgen inhibitors such as abirone acetate(AA), which is poorly absorbed and will stay in the intestine for a long time, and other drugs may produce heterometabolism and change the microflora, thus affecting the efficacy and activity of the drug (27). Daisley et al. found that the biotransformation of AA is affected by *A.muciniphipla.* Oral AA can repeatedly regulate the gastrointestinal microflora associated with patients by promoting the growth of *A.muciniphipla*, while the efficacy of AA is achieved by interacting with *A.muciniphipla* to increase the ability of intestinal microorganisms to synthesize vitamin K2 in PCa patients (175).

**Figure 1:**
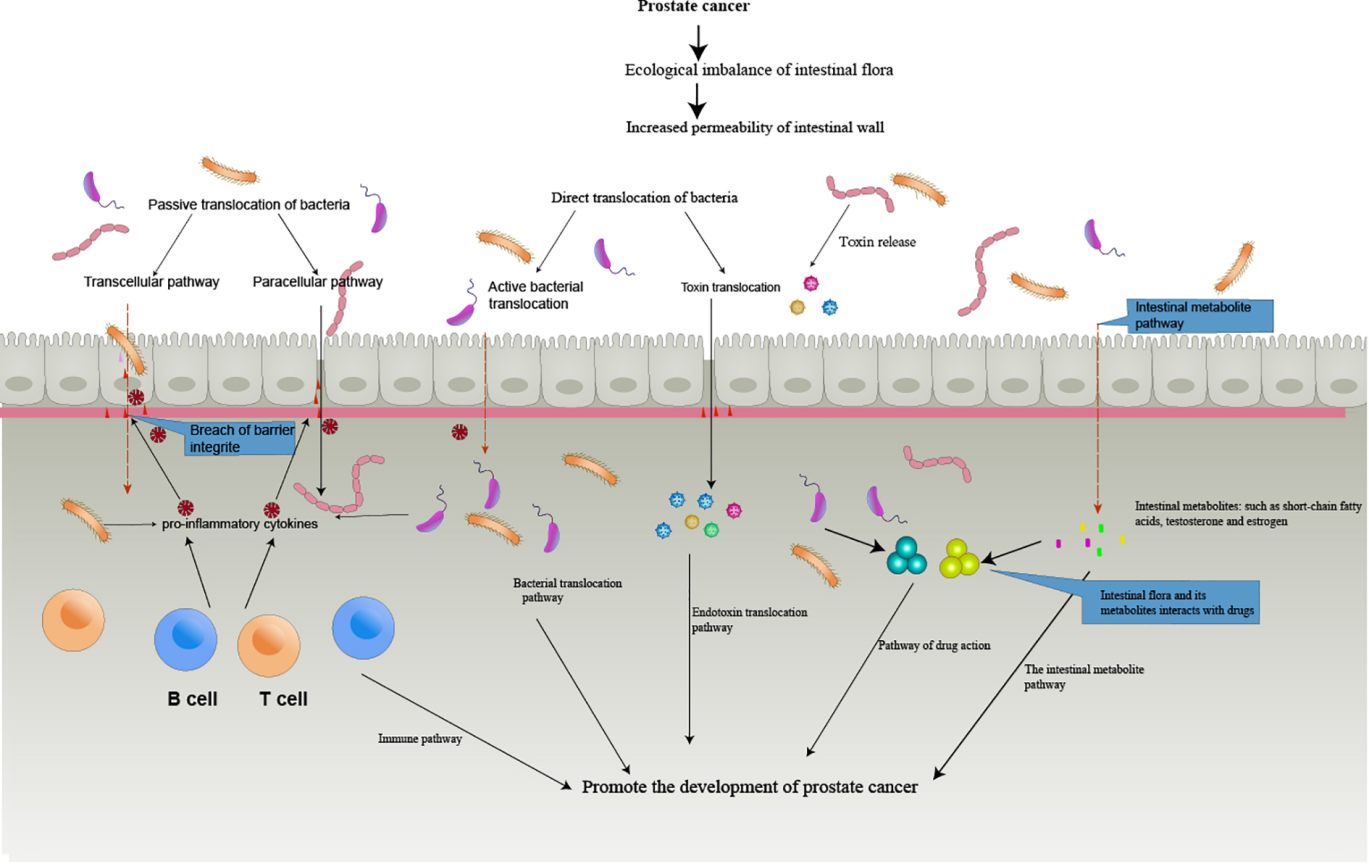
The Intestinal bacterial translocation and toxin translocation. The imbalance of intestinal flora leads to the destruction of the defense mechanism between the intestinal mucosa and the intestinal immune system, and the release of various inflammatory and cytokines destroys the epithelial cell barrier, leading to the active and passive translocation of bacteria, the translocation of toxins, the translocation of bacterial metabolites, and interactions with drugs that eventually enter the circulation, thus promoting the formation of tumors.

Therefore, the further research and development of intestinal microflora may become an important part of personalized and targeted anti-PCa therapy. (**Figure 1**) summarizes the pathway of the gut microbiome in prostate cancer development.

## 3 Treatment

### 3.1 Gut microbiota can be used as a diagnostic tool for PCa

There are more than 1000 species of bacteria in the human intestinal tract, but only 150 to 170 species of bacteria are common to individuals (176). Intestinal microbiota helps to reveal the relationship between complex human diseases, and different diseases may have common and unique characteristics of intestinal microflora (177). Intestinal microflora has been proven to be a tool for non-invasive diagnosis and screening of a variety of diseases, such as liver cancer (178), CRC (179), and gastric cancer (180). In the intestinal microbiota, *Firmicutes* and *Bacteroidetes* accounted for the vast majority, while *Proteobacteria*, *Actinobacteria*, *Verrucomicrobia*, and *the candidate TM7 phylum* were few (181). However, the increase of *Proteobacteria* in the intestinal tract may be a sign of ecological imbalance, and the long-term accumulation of *Proteobacteria* in the intestinal tract is another manifestation of the imbalance of microbial community homeostasis or that the host is in a state of disease (182). A recent study found that *Proteobacteria* may be used as a marker of intestinal microbes in metastatic PCa. The fecal samples of 35 patients with PCa were sequenced by 16S rRNA, and it was found that the abundance of *Proteobacteria* was significantly increased, which was positively correlated with many parameters such as distant metastasis. The relative abundance of *Proteobacteria* was better than the level of prostate-specific antigen in the evaluation of distant metastasis probability of PCa in ROC curve analysis (168). Fruge et al.’s study included 40 overweight men who underwent a radical prostatectomy. Through the microbiological analysis of the 16S rRNA gene in their fecal samples, it was found that the total score of Gleason was positively correlated with *Deferribacteres*, *Proteobacteria*, and *Clostridium*, and negatively correlated with *Blautia (* 183). Matsushita et al. compared the intestinal microbiota of 152 patients with high-grade PCa with that of healthy Japanese men. It was found that 18 kinds of intestinal microorganisms can accurately detect men with high-risk PCa, and their accuracy is higher than the level of prostate-specific antigen, including *Rikenellaceae*, *Alistipes*, *Lachnospira*, *Subdoligranulum*, *Lachnobacterium*, and *Christensenellaceae*, which are significantly increased in high-risk PCa (28).Intestinal microflora, especially *Proteobacteria*, may be a new means of detecting and preventing high-level and metastatic PCa, which is essential for PCa, and more research is needed to explore this aspect.

### 3.2 Metabolites of gut microbiota contribute to the treatment of PCa

#### 3.2.1 Polyphenols

The effects of dietary and nutritional factors on the occurrence and development of PCa have received more and more attention, such as fat and polyphenols (184). The most abundant ingredient in green tea is polyphenols. Intestinal microflora can biotransform dietary polyphenols and improve their bioavailability. Dietary polyphenols can regulate the composition and function of intestinal microorganisms by inhibiting the proliferation of pathogenic bacteria and stimulating beneficial bacteria (16). The proliferation and survival of PCa cells are coordinated by multiple signal pathways (185).Disruption of androgen receptor and PI3K/Akt signal pathways plays an important role in the development of PCa (186, 187), while intestinal microflora can metabolize the main polyphenol epigallocatechin-3-gallate in green tea, reducing the risk of PCa by reducing the influence of androgen receptor (188, 189) and PI3K/Akt signal pathways (190, 191). The relationship between PCa risk and green tea drinking was studied involving 49,920 Japanese men. People who consumed five or more cups of green tea each day had a decreased probability of developing advanced PCa compared to those who drank less than one cup each day (192). In a meta-analysis of nine case-control studies, the risk of PCa was significantly reduced by 57 percent compared with those with the highest green tea intake. Similar results were observed in a recent meta-analysis of 3 case-control and 4 cohort studies, in which the probability of the highest and lowest PCa intake of green tea was significantly reduced by 55% (193). The Ellagitannins (ETs) extracted from pomegranate juice are also a kind of bioactive polyphenol with a chemoprevention effect on PCa. ETs is not completely absorbed in the gastrointestinal tract but is hydrolyzed into different metabolites in the intestine, including ellagic acid, which can be converted into urea A by intestinal microorganisms (194, 195). Several studies have shown that ellagic acid and its microbial metabolite, urea A, can inhibit the growth of PCa cells (196, 197). Cytochrome P1B1 (CYP1B1) is an established target for the chemoprevention of PCa. Too many genotoxic compounds produced by CYP1B1 overexpression might damage normal cells’ DNA and hasten the onset of cancer. Although increased CYP1B1 expression does not result in tumor invasion or metastasis, it can cause anti-PCa medications like flutamide to lose their effectiveness (198). ETs and their microbial metabolites can inhibit CYP1B1 enzyme activity and expression inhibitors, reducing the occurrence and maintenance of PCa (199), causing cell cycle arrest in the G1 phase, and inhibiting whole cell growth (200). All in all, polyphenol-rich diets or compound polyphenol supplements can increase colon metabolites, which in turn contribute to the chemoprevention of PCa (7).

#### 3.2.2 Intestinal lignans

Intestinal flora in the upper large intestine can convert most plant lignans in human food into enterolactone and intestinal diol, called intestinal lignans (EL). Increasing EL intake and enterolactone exposure can reduce the risk of PCa (201). In animal experiments, EL can activate estrogen signal transduction in mice. The ventral prostate is rich in estrogen receptor β. The activation of the estrogen receptor β reduces the epithelial dysplasia of the prostate in mice, which is negatively related to the risk of PCa (202).

#### 3.2.3 Isoflavones

Soybean products usually contain isoflavones, including daidzein and genistein. A number of studies have proven that intestinal flora are involved in the metabolism and biological activity of isoflavones, including *a Clostri-diumsp*, *Eubacterium ramulus*, *Escherichia coli*, *Bacteroides ovatus*, *Ruminococcus productus*, and *Streptococcus intermedius (* 203). Daidzein can induce PCa cell cycle arrest in the G0/G1 phase by affecting the gene expression of cyclin and cyclin-dependent protein kinase (204). Hisae Nakamura provides some experimental data to support the inhibitory effect of isoflavones on the metastasis of prostate tumor cells and suggests that these inhibitory effects may be mediated by ER-β signal transduction (205). A large-scale epidemiological survey of 82483 Hawaiian and Los Angeles men showed that men with the highest legume intake had an 11.1% lower risk of PCa and a 26% lower risk of non-localized or high PCa than those with the lowest legume intake (206). S-equol is a secondary metabolite of daidzein produced by intestinal microorganisms and has stronger anticancer activity than daidzein. Soybean isoflavones are similar to 17β-estradiol in structure. Thus, S-equol can bind to ER and act as a phytoestrogen. ER-β is expressed at a higher level in prostate tissue,the binding affinity of S-equol to ER-β is similar to that of 17β-estradiol, but stronger than that of ER-α, and soy isoflavones are more likely to induce ER-β transcriptional activity (207). Hirokazu Tsuji et al. recently discovered a new intestinal bacteria, *Slackia* sp. *Strain NATTS*, which belongs to the Slackia genus and can quickly degrade daidzein into S-equol (208). According to a study of 14203 Japanese men conducted by Kurahashi et al., the highest tertile of plasma S-equol was significantly associated with a reduction in total PCa risk, especially in local cancers (209). S-equol inhibits the growth of LNCaP, DU145, and PC3 cells of human PCa by up-regulating the expression of Forkhead box O3, a tumor-suppressing transcription factor in PCa (113). Among 28 healthy volunteers in Japan, 18 S-equol producers and 10 S-equol non-producers took soybean isoflavones for three months. Three months later, it was found that there was no significant change in serum estradiol and total testosterone levels, while serum sex hormone binding globulin levels significantly increased and serum free testosterone and dihydrotestosterone levels decreased significantly. Long-term soy isoflavone supplementation can stimulate the production of serum estradiol and reduce serum dihydrotestosterone levels in men, thereby reducing the risk of PCa (210). In a large population study, people who ate soy milk more than once a day were associated with a 70% lower risk of PCa, and serum phytoestrogens (genistein, daidzein, and S-equol) had dose-dependent protective effects on the development of PCa (211). Therefore, H. Akaza proposed that S-equol is a key factor in the difference in incidence between Asia and the West. PCA may be susceptible due to a lack of S-equol-transforming bacteria in the intestinal environment and an inability to convert daidzein into S-equol. S-equol-containing supplements can be used to improve the intestinal environment and prevent the occurrence of PCa (212).

#### 3.2.4 Indole-3-methanol

Cauliflower, cabbage, and cauliflower all belong to the same family of cruciferous vegetables, which are significantly associated with the occurrence of PCa. Eating three or more servings of cruciferous vegetables per week can reduce the risk of PCa by 40% (213). Indole-3-methanol (I3C), a derivative of cruciferous vegetables, can derive a series of metabolites in an acidic gastrointestinal environment and prevent PCa. The addition of I3C significantly inhibited tumor growth (p < 0.0001) and changed the structure of the intestinal microbiome, in which the specific bacterial group (*M.schaedleri*) of *Deferribacteres* increased significantly. I3C also destroys the interaction between microorganisms, and its chemoprevention may be related to the changes in the composition of microflora and microbial interaction in the intestinal microbial community (214).

In conclusion, the supplementation of beneficial bacteria which produce polyphenols, EL, isoxanthol and indole-3-carbinol significantly reduced the risk of cancer. At present, dietary supplements are the simplest means to regulate the microbiota, and dietary pattern intervention may help to prevent PCa by changing the microbiota. Exploration of more beneficial microbiota is the key to understanding the influence of diet and nutrition on PCa.

### 3.3 Probiotics intake

Probiotics are crucial for maintaining the composition of intestinal microbiota and enhancing the equilibrium of intestinal microflora. By boosting mucosal barrier function and antibody production, boosting epithelial integrity, and preventing the entry of harmful microbes, they can enhance the host immune response (215, 216). The only probiotic strain that can compete with *Escherichia coli*, which causes chronic inflammation, is *EcN* (*Escherichia coli Nissle 1917-EcN*). Oral probiotics can alter the intestinal microbiota and have an impact on the prostatic inflammatory milieu, according to a new study by Manfredi et al. Two groups of patients with persistent bacterial prostatitis were randomly assigned. Levofloxacin was initially administered to all subjects, and on the basis of this, oral *EcN* was administered to the experimental group. The biological recurrence rates after 3 months and 6 months considerably lowered in the experimental group, and patient adverse responses were infrequent throughout the experiment. the combination of *EcN* with levofloxacin can better control the symptoms and biological recurrence in individuals with chronic bacterial prostatitis without reducing the safety of treatment (217).

In another study, mice fed foods rich in the probiotic strain *L. reuteri* could reduce systemic inflammation by reducing IL-17A and increasing serum testosterone levels and supplement *L. reuteri* or other probiotic supplements to prevent male hypogonadism, which may give individuals healthier reproductive hormones and gonadal characteristics (218). Anti-cancer immunotherapy will be more effective if specific intestinal resident flora are used. *Lactobacillus rhamnosus GG* (LGG), the most studied probiotic model of cancer, can be observed in animal models to have anti-inflammatory effects and promote tumor regression (219). In addition, cancer patients lack natural B vitamins, and folic acid and arginine have the greatest metabolic changes (25), Through the use of probiotics and the elimination of external supplements, natural folic acid production may increase in high-risk men, and external sources may increase cancer risk (25). *Bifidobacterium* can improve self-anti-tumor immunity by promoting the efficacy of antibodies on the axis between programmed cell death protein 1 and its ligand (164). Butyrate is an anti-inflammatory micronutrient produced by *Faecalibacterium prausnitzii* and *Eubacterium rectalie* in the intestinal tract and may be related to one of the ways to prevent PCa (7). Tumor necrosis factor-related TRAIL is an endogenous cytokine that can induce apoptosis in malignant tumor cells. Treatment with *Lactobacillus* can also antagonize PCa by producing TRAIL in peripheral blood mononuclear cells to promote NK activity (220). A recent study has found that intravenous injection of extracellular vesicles derived from *A.muciniphipla* in mice can establish a CD8+ cytotoxic T cell response to mouse PCa in immunoactive C57BL/6 mice and slow tumor progression in the absence of ADT (221). Probiotics do play a role in tumor prevention and treatment, and treatment with probiotics may be an economical and convenient anti-cancer strategy in the future.

### 3.4 Fecal microbiota Transplantation (FMT)

FMT can regulate intestinal microbiota to improve the response rate of immunotherapy-resistant patients (222). FMT is an effective method to transplant the intestinal microbiota of healthy donors into patients to restore intestinal microbiota diversity, which is generally treated through the upper or lower digestive tract (223). In the management of *Clostridium difficile* infection, FMT has demonstrated notable efficacy. FMT was approved as a clinical approach for the treatment of recurrent *Clostridium difficile* infection in the 2013 guidelines (223). Sivan et al. first revealed that intestinal microbiota may play a role in regulating anti-tumor immunity. They carried out FMT on melanoma mice with different intestinal microbiota contents and found that the tumor growth rate was surprisingly similar, and the tumor growth rate of the side with rapid tumor progress was significantly reduced after FMT (164). Baruch et al. designed a phase I clinical trial to perform FMT on 10 patients with malignant melanoma resistant to anti-PD-1 therapy, including 1 patient with complete remission and 2 patients with partial remission. Furthermore, both their intestinal lamina propria and tumor microenvironment show improved signals for gene spectrum and immune cell infiltration (224). Riquelme et al. used 16S rRNA gene sequencing to analyze the composition of tumor microbiota in pancreatic cancer patients. Long-term survivors of *Pseudoxanthomonas*, *Saccharopolyspora*, *Streptomyces*, and α-diversity outnumber short-term producers. They transplanted the fecal flora of patients with advanced pancreatic cancer with a survival period of > 5 years into mice, and the number of CD8+ T cells in the mice increased significantly, and their tumors shrank significantly. They speculated that FMT changed the diversity and structure of the tumor microenvironment flora to promote the immune response in the mouse model (225). Liu et al. found that the intestinal flora transplantation using CRPC feces accelerated the progress of PCa in mice and increased the abundance of *ruminococcus* related to poor prognosis, which proved that FMT did have an effect on PCa (60). Androgen deprivation is very important in the treatment of PCa, but almost all patients will be resistant. The FMT treatment is not complicated. FMT updates intestinal microbiota to reduce drug resistance in patients and carries out personalized bacterial transplantation, which may be a favorable weapon for PCa treatment in the future.

## 4 Conclusions

We summarized the different gut microbiota compositions of a large number of prostate cancer patients compared with non-prostate cancer patients and found that certain gut microbes were associated with an increased risk of prostate cancer, including (*Bacteroides massiliensis*, *Bacteroides*, *Streptococcus Tissierellaceae*, *Lachnospiraceae*, *Pseudomonadales*, *Proteobacteria*, *Gammaproteobacteria*, *Ruminococcus*, *Bacillus*, *Rikenellaceae*, and *Clostridiales*), and other organisms. Gut metabolites such as estrogen, androgen, folate, and short-chain fatty acids are involved in the pathogenesis of prostate cancer, while their other metabolites such as polyphenols, ETs, and isoflavones are helpful for the treatment of prostate cancer. It seems that intestinal microorganisms can directly affect PCa through bacterial translocation or toxins and may also be indirectly affected by intestinal microbiota, including metabolites, immunity, genes, and the effects of anti-tumor drugs. If PCa is confirmed to be affected by intestinal microbiota, changing intestinal microbiota through diet or FMT treatment will provide an effective treatment strategy for PCa prevention and treatment. Certain microorganisms may also be used as diagnostic markers of PCa. In a word, the intestinal microbiome may play an important role in the development, diagnosis, and treatment of PCa. More research is needed to explore the complex interrelationships and ways of action of the intestinal microbiome. More research is needed to understand the role of the intestinal microbiome in PCa patients, and personalized treatment is used to target the intestinal microbiome of different patients.

## Author contributions

KT and QW conceptualized the review, analyzed the data, and helped to write the manuscript. CZ, KH, ZP, LZ, WL, BC, SX, YY, TH, and WZ helped to write the manuscript and prepared the figures. All authors contributed to the article and approved the submitted version.
